# A Complicated Labour

**Published:** 1886-03

**Authors:** John D. Skeer

**Affiliations:** 681 Washington Boulevard


### A COMPLICATED LABOUR. By DR. JOHN D. SKEER.

[Read before the Chicago Pathological Society, February 8th, 1886.]

Mrs. H., æt. 27, American, primipara. Labour began at one A. M. on September 1st, 1885, and at seven A. M. her family physician, Dr. E. Wyllys Andrews, was sent for. On arriving he found the os uteri slightly dilated, but could, however, make out a vertex presentation. Labour progressed from that time naturally, but slowly, until 3:15 P. M. of the same day, at which time a living, well-formed child was born. Immediately Doctor Andrews discovered the presence of a second child in the cavity of the uterus. The pains had ceased, and after waiting a half-hour, a small dose of the fluid extract of ergot was administered, which was repeated at the end of an hour. These methods failed to excite the slightest uterine contractions. At 5:30 P. M. I was called in consultation.

We found it very difficult to make out the presentation, which proved to be the tuberosity of the right ischium, occupying the middle of the superior strait. The child's pelvis was tipped toward the mother's left side. An undetached placenta occupied the left iliac fossa, extending nearly to the os uteri.

As there was no hæmorrhage, we waited an hour and gave more ergot, but the pains continued to be irregular and inefficient, and we decided to bring down the feet and deliver at once. On introducing the hand, but one foot and one arm could be felt, the latter inclined to fall down in the plevis. In the position that should have been occupied by the left leg and arm a large fleshy mass could be felt without any definite outline. The right leg was brought down with the toes to the front, and traction was made without causing the slightest change in the position of the child—it was firmly fixed, and held as though in a vise. Strong, unremitting traction for about half an hour, at the same time rotating the child toward the mother's left side, brought the child's pelvis through the vulva with the heel to the front. The child was then delivered as in ordinary breech presentations, and was found to be well developed, dead, and not a monstrosity as was at first supposed.

As the placentæ were not expelled within an hour, the cords were traced upward; one led to the lower placenta, the other was traced upward through a small opening beyond our reach. We then realized for the first time that we had an hour-glass contraction of the uterus, which had taken place after the delivery of the first child. With careful bi-manual palpation the constriction was overcome, and the upper after-birth removed. It was adherent throughout its entire extent. Immediately after the removal of the upper placenta the lower one became detached, and was readily brought away. The placentæ were united by a strong fibrous band about six inches in width. The patient made a good recovery, and nothing occurred in the after-treatment worthy of note.

Conclusions: Regarding the position of the second child, we had, below the constriction, the body, the right arm and leg, and, as it proved, the left thigh and leg. For on no other hypothesis can we account for the oblique position of the pelvis, unless the foot were grasped and held in the upper chamber by the contraction. The left thigh and leg were behind and completely covered by the membranous band uniting the placentæ, so that their position could not be determined. Rotating the child towards the mother's left slipped the left thigh and leg from behind the band, without detaching the after-birth. Had this latter accident happened hæmorrhage would have been a serious, if not fatal, complication.

681 Washington Boulevard.

